# Functional compounds of ginseng and ginseng-containing medicine for treating cardiovascular diseases

**DOI:** 10.3389/fphar.2022.1034870

**Published:** 2022-12-02

**Authors:** Lanchun Liu, Jun Hu, Qiyuan Mao, Chao Liu, Haoqiang He, Xiaoshan Hui, Guang Yang, Peirong Qu, Wenjing Lian, Lian Duan, Yan Dong, Juhua Pan, Yongmei Liu, Qingyong He, Jun Li, Jie Wang

**Affiliations:** ^1^ Departmen of Cardiology, Guang’anmen Hospital, China Academy of Chinese Medical Sciences, Beijing, China; ^2^ Departmen of Oncology, Guang’anmen Hospital, China Academy of Chinese Medical Sciences, Beijing, China; ^3^ Beijing University of Chinese Medicine, Beijing, China

**Keywords:** panax ginseng, ginsenoside, herbal medicine, functional components, cardiovascular disease

## Abstract

Ginseng (Panax ginseng C.A.Mey.) is the dry root and rhizome of the Araliaceae ginseng plant. It has always been used as a tonic in China for strengthening the body. Cardiovascular disease is still the main cause of death in the world. Some studies have shown that the functional components of ginseng can regulate the pathological process of various cardiovascular diseases through different mechanisms, and its formulation also plays an irreplaceable role in the clinical treatment of cardiovascular diseases. Therefore, this paper elaborates the current pharmacological effects of ginseng functional components in treating cardiovascular diseases, summarizes the adverse reactions of ginseng, and sorts out the Chinese patent medicines containing ginseng formula which can treat cardiovascular diseases.

## Introduction

Cardiovascular-related disease still remains a major public health problem with high morbidity and mortality in some countries. According to the “Global Health Estimates Report 2022” released by WHO (World Health Statistics, 2022), due to population growth and longer life expectancy, the total number of deaths from non-communicable diseases has increased, and about 33.2 million people worldwide died from cancer, cardiovascular disease, diabetes and chronic respiratory diseases. The “China Cardiovascular Health and Disease Report 2021 Summary” also pointed out that the prevalence and mortality of cardiovascular diseases in China are still on the rise, cardiovascular disease in rural and urban areas accounted for 46.74% and 44.26% of the cause of death respectively, and the burden is still increasing (Chinese Cardiovascular Health and Disease Report Writing Group, 2022). According to a 2020 report by the American Heart Association (AHA), cardiovascular disease kills about 850,000 people and costs more than $300 billion a year, and its main risk factors are high cholesterol, smoking, and diabetes (Virani et al., 2020).

Relevant studies have shown that the occurrence and development of cardiovascular disease CVD is closely related to a variety of pathological processes. For example, coronary artery disease (CAD) is caused by cholesterol deposits and inflammation that damage the coronary arteries, leading to atherosclerosis which results in insufficient oxygen and blood supply. The pathological mechanism depends on dyslipidemia (Borén et al., 2020), inflammation of blood vessels (Ahluwalia et al., 2013), disorder of endothelial function (Davies et al., 1993; Gutiérrez et al., 2013), infiltration and turnover of macrophages (also known as macrophage polarization) (Stöger et al., 2012), etc. Platelets can cause blood clot problems and play an important role in a variety of cardiovascular diseases. During myocardial ischemia, chemicals such as adenosine, bradykinin, lactate, reactive oxygen species (ROS) and histamine are released to stimulate nerve receptors and produce angina symptoms, while the ischemia-reperfusion period will aggravate the accumulation of ROS and cause DNA damage, leading to cardiomyocyte apoptosis (Rezende et al., 2019). Hypertension is emerging as one of the major risk factors for cardiovascular and renovascular disease and also becoming a major risk factor for death worldwide (Ezzati et al., 2002). The main pathological links involved in the occurrence of hypertension include increased endothelin (ET), decreased nitric oxide (NO)/nitric oxide synthase (NOS), and imbalance of renin-angiotensin-aldosterone system (Zhang, 2011). Endothelial dysfunction, arginase activation, decreased NO bioavailability, and increased vascular stiffness are also important factors in leading hypertension (Ryoo et al., 2011; Konukoglu and Uzun, 2017). Another study found that essential hypertension is closely related to an increase in ROS and cell death mediated by defective mitochondrial oxidative phosphorylation (Zhang et al., 2014). Cardiac Arrhythmia is a common cardiovascular disease in clinic. It is a group of disorders that cause abnormal heart beat frequency and/or rhythm due to the origin conduction disorder of cardiac electrophysiology. Arrhythmias is classified as impulse formation abnormality, impulse conduction abnormality, tachyarrhythmias and bradyarrhythmias (Liu, 2021). There are 3.7 million sudden cardiac deaths in the world every year, and a considerable part is caused by severe arrhythmia (Kuriachan et al., 2015). According to the epidemiological survey of sudden cardiac death in China, more than 80% of sudden cardiac death events are caused by malignant arrhythmia every year (Chen et al., 2020). Heart failure (HF) is a serious public health problem and the leading cause of death from cardiovascular disease worldwide. It is a complex clinical syndrome caused by abnormal changes in cardiac structure and function due to various reasons, resulting in dysfunction of ventricular systolic and diastolic functions which mainly manifesting as dyspnea, fatigue, and fluid retention (Heart Failure Group of Cardiology, 2018). According to the “China Cardiovascular Disease Report 2020,” there are 8.9 million heart failure patients in China, The China Heart Failure Patient Registration Study (China-HF) shows that the mortality rate of hospitalized patients with heart failure is 4.1% (China Cardiovascular Health and Disease Report Writing Group, 2021), the 5-year survival rate of heart failure patients is comparable to that of malignant tumors (Kannel, 2000). However, with the wider application of drug therapy, patients can benefit from it, but at the same time, some toxic and side effects have gradually become prominent. For example, long-term use of lipid-lowering drugs will have adverse reactions of myopathy and liver. Aspirin and warfarin often cause gastric mucosal damage, severe intracranial hemorrhage, and even death. Calcium channel blocker (CCB) can cause blurred vision and eye pain. Diltiazem can cause auditory hallucinations and vision loss in patients. Verapamil can cause vertigo symptoms (Yi, 2013). Side effects of antihypertensive drugs include gastrointestinal reactions, nervous system toxicity, etc. Antiarrhythmic drugs themselves also have proarrhythmic effects (Liao and Yang, 2008).

The World Health Organization defines the functional components of herbal medicines as “the components that have a therapeutic effect in herbal medicines.” Traditional Chinese Medicine (TCM) has played an important role in preventing cardiovascular diseases by applying herbal medicine based on its unique theory and experience (Hao et al., 2017). Several preclinical studies have confirmed that ginseng and its main functional components are involved in the treatment and prevention of cardiovascular disease, and have the potential to reduce cardiovascular risk factors. From 1976 to 1978, Chinese researchers isolated and identified three saponins from ginseng, namely panaxadiol, panaxatriol and oleanolic acid saponins (Li and Teng, 1978; Chen et al., 2002). Subsequently, ginseng polysaccharides, volatile oil and other components were continuously explored. This article aims to clarify the pharmacological mechanism of ginseng functional components in the prevention and treatment of cardiovascular disease, and to sort out the clinical application of ginseng’s formulation in detail.

## Pharmacological effects of ginsenosides in treating cardiovascular diseases

**FIGURE 1:**
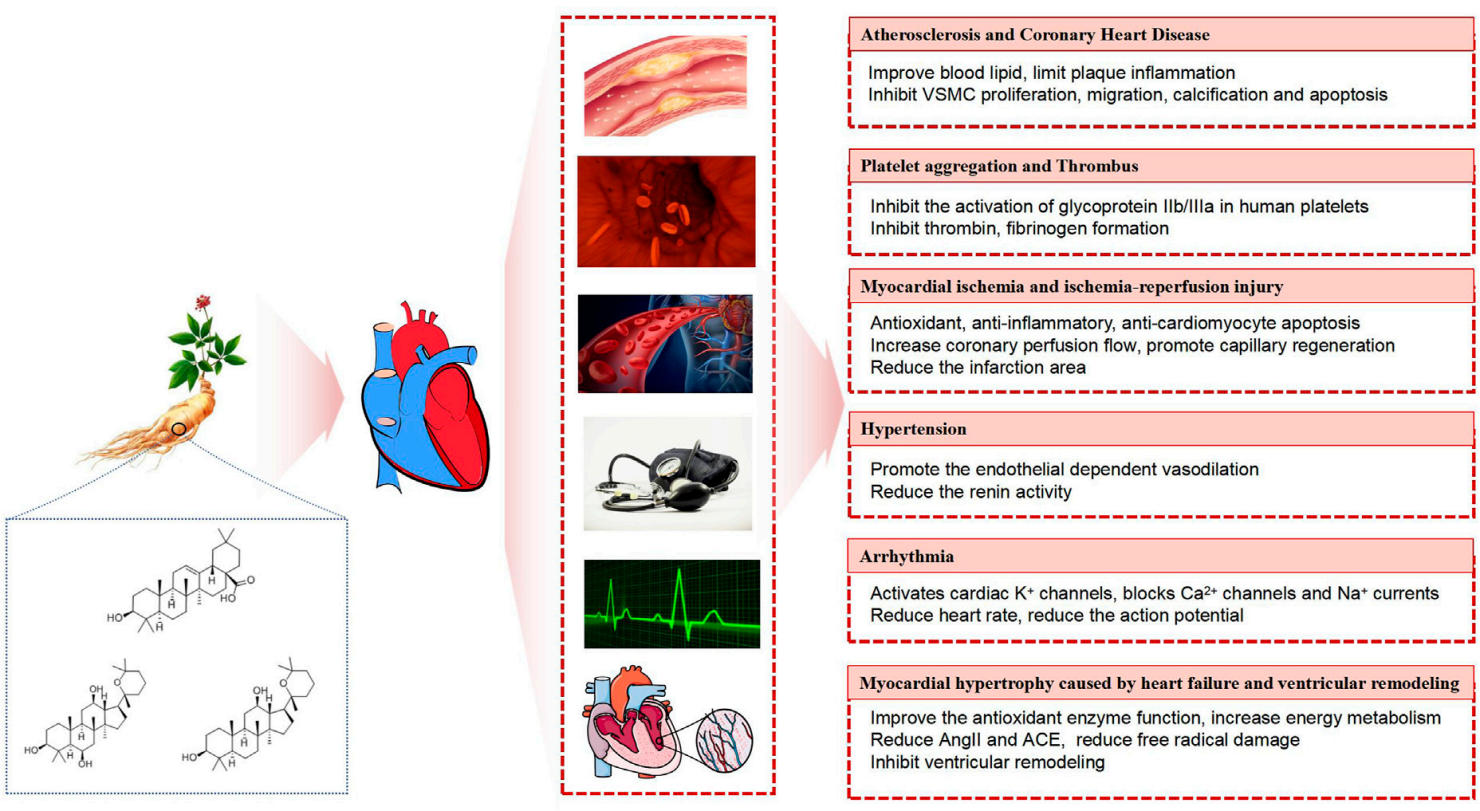
Ginsenosides in treating cardiovascular diseases.

Ginsenosides belong to triterpenoid saponins and are mainly divided into three types: protopanaxadiol type, such as ginsenoside Ra1, Rbl, Rb2, Rc, Rd, etc. protopanaxatriol type, such as ginsenoside Re, Rf, Rgl, Rg2, Rhl, etc. Oleanolic acid type, such as ginsenoside RO, Rh3, etc (Chu and Zhang, 2009; Cao et al., 2012) (Figure 1). In one study, a variety of ginsenosides at different sites of ginseng were analyzed using ultra performance liquid chromatography-quadrupole time of flight /mass spectrometry (UPLC-QTOF/MS), and then multivariate analysis was performed on the dataset, according to the results, ginsenosides such as Ra1 and Ra2, are unique among the roots of ginseng (Lee et al., 2017a).

### Atherosclerosis and coronary heart disease

Ginseng has shown to involve in the atherosclerotic gene regulation with anti-inflammatory effects and results in the changes in improving in lipid profile (Duan et al., 2017; Cholesterol Treatment Trialists' (CTT) Collaboration O'Connell et al., 2015). Studies have found that protopanaxadiol and protopanaxatriol saponins can improve the lipid profile by reducing the concentrations of cholesterol, triglycerides, low-density lipoprotein and free fatty acids in plasma, while increasing the total concentration of high-density lipoprotein (Deng et al., 2017). This lipid metabolism-modulating effect of ginseng supplementation was also validated in a meta-analysis (Hernández-García et al., 2019). Ginsenosides can also reduce the progression of atherosclerosis by inhibiting the expression of VCAM-1 and enhancing α-smooth muscle actin (α-SMA) (Zhang et al., 2004). In addition to improving blood lipid levels, ginseng extract can also reduce superoxide dismutase (SOD) and catalase (CAT) levels (Kim and Park, 2003). Studies have shown that ginsenoside compounds K and Rb1 are potential active components to restore TNF-α-induced oxidative stress and oxidized low-density lipoprotein (ox-LDL)-induced inflammation and apoptosis (Zhou et al., 2017a; Lu et al., 2019). In animal models, ginsenosides Rg3 and Rb1 are thought to inhibit vascular smooth muscle cell (VSMC) proliferation, migration, calcification, and induce apoptosis (Guo et al., 2018a; Nanao-Hamai et al., 2019). Ginsenoside Re (G-Re) can affect platelet-derived growth factor-BB (PDGF-BB)-induced proliferation of VSMCs by regulating the eNOS/NO/cGMP pathway through G0/G1 cell cycle arrest (Gao et al., 2019). Another study found that ginsenosides Rb1 and Rg1 could inhibit the apoptosis process in atherosclerosis model by increasing autophagy (Zhou et al., 2018; Yang et al., 2018).

Ginseng can also reduce atherosclerosis, or limit intraplaque inflammatory responses by modulating macrophage polarization in order to prevent the occurrence of atherosclerosis (Guo et al., 2018b; Zhang et al., 2018). Recently, a study has proposed that the anti-inflammatory effects of several ginsenosides (Rg3, Rb1, and Rg1) may be due to inducing M2 polarization of macrophages and microglia, which in turn helps to inhibit inflammatory progression and promote inflammation resolution (Im, 2020). By inhibiting the expression of NF-ĸB and JNK, ginsenosides can not only reduce the production of inflammatory cytokines such as VCAM-1 and ICAM-1, but also reduce the number of macrophages, thereby controlling the size of atherosclerotic lesions (Su et al., 2016). A study has shown that ginsenosides can reduce the expression of interleukin, inhibit the expression of NF-ĸB/p65, and exert anti-inflammatory effects (Sun et al., 2013; Zhang et al., 2008). Ginsenoside Rg3 can reduce the expression of cell adhesion molecules and pro-inflammatory cytokines in blood vessels, inhibit the expression of TNF-α, and has anti-inflammatory and anti-atherosclerotic effects (Hien et al., 2010). Another study found that ginsenoside Rb1 can significantly inhibit inflammation, oxidative stress and apoptosis by inhibiting the production of ROS and MDA, reducing the expression levels of IL-6, IL-1, ICAM-1, VCAM-1, VEGF, MMP-2, and MMP-9 (Zhou et al., 2017b). Based on the above research results, it can be found that ginseng has a good effect in treating and preventing atherosclerosis and coronary heart disease caused by inflammation and lipid profile.

### Platelet aggregation and thrombus

The existing research results show that ginsenosides can exert antiplatelet and anticoagulant activities through various mechanisms. Ginsenoside Rg1 was found to improve the aggregation of platelets and formation of arterial thrombus, which may be achieved by regulating the ERK/Akt signaling pathway (Zhou et al., 2014). What’s more, ginsenoside Rg3 has also been found by two other studies to inhibit the platelet aggregation process through the key signaling pathways such as ERK2 and cAMP (Lee et al., 2008; Kwon, 2018a). Ginsenoside Rg3 can also achieve antithrombotic effect by inhibiting the formation of thrombin (Jeong et al., 2017). For 20(S)-ginsenoside Rg3, its antiplatelet mechanism may be related to the inhibition of glycoprotein IIb/IIIa activation in human platelets (Kwon, 2018b). In another study, ginsenoside Ro was shown to inhibit the binding of fibrinogen to αIIb/β3 in human platelets (Shin et al., 2016). A study evaluated the synergistic effects of different ginsenosides to assess whether structural modifications affect their antiplatelet activity. Ginsenoside Rp3 was prepared from the structural modification of Re by means of reduction with hydrogenation. Rp1 was prepared from other ginsenosides (Rg3, 2h-Rg3, Rg5, and Rk1) with the same method that was used for Rp3 production. 2H-Rg3 is called Dihydroxy-G-Rg3, which is chemically derived from Rg3 by means of reduction with hydrogenation. It was found that G-Rp3 was shown to be effective in inhibiting platelet aggregation by exerting synergistic effects with G-Rp1 and 2H-Rg3 (Irfan et al., 2020). Therefore, the research on ginsenosides will help to develop new antiplatelet and antithrombotic drugs in the cardiovascular system.

### Myocardial ischemia and ischemia-reperfusion injury

Ginseng total saponin has antioxidant and anti-inflammatory effects, so it can inhibit oxidative stress and reduce myocardial damage (Aravinthan et al., 2015). For example, the combination of ginsenoside Rb3 and Rb2 has a protective effect on myocardial ischemia-reperfusion injury, and its mechanism may be related to anti-inflammatory response, improve oxidative stress and resist cardiomyocyte apoptosis (Liu et al., 2020a). Studies have found that ginsenoside Rbl can reduce serum aspartate transaminase (AST), Lactic dehydrogenase (LDH) and creatine kinase in myocardial tissue, protect the heart by resisting inflammatory and apoptotic damage (Zheng et al., 2017a). Ginsenoside Rg3 also has similar efficacy, which may be achieved by regulating Akt/eNOS and Bcl/BAx signal transduction pathways (Wang et al., 2015; Zhang et al., 2016). Ginsenosides can protect cardiomyocytes from hypoxia/reoxygenation damage by reducing oxidative damage (Li and Liu, 2006), which may be related to reducing intracellular calcium overload (Xu et al., 2005) and inhibiting the activation of JNK signaling pathway (Li et al., 2012; Li et al., 2017; Sun et al., 2019). Ginsenoside Rg1 can also inhibit autophagy in H9c2 cardiomyocytes (Zhang et al., 2012), improve mitochondrial dynamics imbalance (Dong et al., 2016), and promote capillary regeneration in ischemic myocardial tissue (Wang et al., 2005; Zhang and Liu, 2009). Ginseng can also activate the reperfusion injury salvage kinase (RISK) signaling pathway through glucocorticoid receptor/estrogen receptor (GR/ER) in an endothelium nitric oxide synthase (eNOS)-dependent mechanism (Zhou et al., 2011). Another study stated that ginseng can enhance the PI3K/Akt/eNOS pathway, increase the coronary perfusion flow of the heart, and reduce the infarction size (Yi et al., 2010). It is worth mentioning that ginsenoside Rc as well as Rb1 and Re can also inhibit coronary vascular dysfunction (Chai et al., 2005).

### Hypertension

There is substantial evidence that ginsenosides are beneficial in the treatment of hypertension, not only does ginseng lower blood pressure, it also acts as a heart protector (Jeon et al., 2000; Nagar et al., 2016). A study shows that ginsenoside Rb3 can reduce oxidative stress in hypertension and protect endothelial function (Wang et al., 2014). In a spontaneously hypertensive rat model, Rg1 has protective effects not only in large arteries but also in small resistance arteries (Chen et al., 2012). Ginsenosides have been found to inhibit arginase, stimulate endothelial nitric oxide synthase coupling, block homocysteine-induced ROS damage, promote endothelium-dependent vasodilation, and achieve the purpose of lowering blood pressure (Shin et al., 2013; Zhou et al., 2005). In addition to the above studies, ginsenoside Rg3 can reduce renin activity by stimulating the expression of iNOS/NO, and also reduce blood pressure (Lee et al., 2016).

Ginseng has been shown to lower blood pressure in several studies, but there have also been reports of raising blood pressure, which may be related to the bidirectional effect. In previous literature, a study found ginsenosides can cause biphasic changes in blood pressure without affecting breathing and heart rate (Takagi et al., 1972). Another study found that large doses of ginsenoside Rb1 can cause an increase in blood pressure, and all other ginsenosides except Rb1 showed biphasic changes (Kaku et al., 1975). Although the effect of ginseng in blood pressure has had conflicting results in previous studies, a recent systematic review of randomized, double-blind, placebo-controlled trials has preliminarily resolved the inconsistencies in ginseng evidence for blood pressure regulation, the results of this study provide optimistic evidence for the efficacy of ginseng in reducing prehypertension, acute hypertension and chronic hypertension (Lee et al., 2017b).

### Arrhythmia

Ginsenosides can affect the electrophysiology of cardiomyocytes and are used to regulate arrhythmias. Among them, ginsenoside Re is a major phytosterol of ginseng, which can activate the K^+^ channel of the heart through the non-genomic pathway of sex hormones (Furukawa et al., 2006), and can also block the Ca^2+^ channel, reduce the heart rate, reduce the action potential plateau phase, and reduce the P wave amplitude (Jin and Liu, 1994). Several other studies have found that ginsenoside Re can regulate K^+^ and Ca^2+^ currents in cardiac electrical activity by inducing NO and cyclic guanosine monophosphate pathways (Bai et al., 2003; Bai et al., 2004; Choi et al., 2009). Ginseng Rg2 has been studied in rat models of calcium chloride-induced arrhythmias, and it has been found to have anti-arrhythmic effects, including shortened duration, mortality, and incidence of malignant arrhythmias, which may be related to inhibition of phosphorylation of Ca^2+^ (Gou et al., 2020). Experiments have shown that the use of ginsenosides to intervene in arrhythmias can increase the amplitude of the T wave and reduce the amplitude of the QRS wave, thereby restoring the heart rhythm to normal (Chen and Zhang, 2009; Lu et al., 2012). A study found that ginsenoside Rg3 can change the electrocardiogram (ECG) and monophasic action potential (MAP) of Langendorff perfused rabbit heart, shorten the QT interval, and may be related to alleviating the current inhibition of human ether-related genes (hERG) and accelerating the activation process of potassium channels (Zhang et al., 2021). Another study has shown that ginsenosides can also treat arrhythmias by inhibiting the voltage-dependent Na^+^ current in the myocardium and reducing the amplitude of action potentials (Liu et al., 2019). These studies fully demonstrate the antiarrhythmic therapeutic potential of ginsenosides.

### Cardiac hypertrophy due to heart failure and ventricular remodeling

Heart failure is a kind of disease that the impaired heart function or structure causes decreased ventricular filling or decreased ejection function, and the heart output is not enough to meet the needs of the body. There are many causes of heart failure, mainly including the excessive pressure overload during cardiac systole, the excessive volume overload during cardiac diastole, the abnormal energy metabolism of cardiomyocytes, the use of cardiotoxic drugs, and the myocardial fibrosis or ventricular remodeling (Lin et al., 2017).

In improving cardiac systolic and diastolic function, a study has found that ginsenoside Rg3 can significantly inhibit the proliferation of middle vascular smooth muscle cell proliferation, reduce stromal hyperplasia, and enhance vasodilation and vasoconstriction function in elderly rats (Liu et al., 2016). It also has found that ginsenosides can enhance the systolic and diastolic functions of the left ventricle after heart failure (Wang et al., 2008). In terms of regulating the energy metabolism, ginsenoside Rb3 can inhibit mitochondria-mediated apoptosis, upregulate energy metabolism, activate fatty acid oxidation, and exert cardioprotective effects (Chen et al., 2019). The active ingredients of ginseng also have a certain therapeutic effect on heart failure caused by cardiotoxicity. For example, ginsenoside Re can improve myocardial fibrosis and heart failure induced by isoproterenol in rats (Wang et al., 2019). It was found that the expression of p-P70S6K, c-Jun N-terminal kinase 1 and Beclin1 decreased in the ginsenoside Rg1 group. These results showed that ginsenoside Rg1 can reduce the expression of doxorubicin-induced cardiac microtubule-associated protein-light chain 3 and autophagy-related 5, reduce doxorubicin-induced endoplasmic reticulum dilation, and improve cardiac insufficiency by inhibiting endoplasmic reticulum stress and autophagy (Xu et al., 2018). Another study found that ginseng can treat adriamycin-induced heart failure, increase the activity of myocardial glutathione peroxidase (GSH-Px), alleviate mitochondrial damage, reduce the production of ROS, and reduce the amount of ascites (You et al., 2005). In addition, ginsenoside Rbl can improve cardiac function and remodeling in patients with heart failure, and the mechanism may be that ginsenoside can not only reduce β-myosin heavy chain (βMHC), angiotensin I converting enzyme (ACE), angiotensin II (AngII) and atrial natriuretic factor (ANF), but also regulate mitochondrial membrane potential (Zheng et al., 2017b). In addition, the active compounds of ginseng can reduce myocardial hypertrophy and oxidative stress (Tsai et al., 2014), and inhibit cardiac fibrosis and heart failure (Guo et al., 2011; Lo et al., 2017). Some studies have also found that ginsenosides can effectively inhibit the right ventricular hypertrophy induced by monocrotaline in rats, suggesting that it has an anti-ventricular hypertrophy effect (Jiang et al., 2007). This effect has also been found to be dependent on phospho-akt (p-akt) activation and inhibition of p38 mitogen-activated protein kinase (MAPK) (Zhang et al., 2013a), and is often associated with inhibition of vascular mitogenic activity (Qin et al., 2008), both of which have been experimentally verified *in vitro* and *in vivo* (Zhang et al., 2019). The contents of functional compounds of ginsenoside are summarized in Table 1.

**TABLE 1:** Chemical structure classification of ginsenoside components.

Name	Component or compound	R1	R2
Protopanaxadiol ginsenosides PPD	Rb1	Glc (2-1)Glc	Glc (6-1)Glc
	Rb2	Glc (2-1)Glc	Glc (6-1)Arap
	Rb3	Glc (2-1)Glc	Glc (6-1)Xyl
	Rc	Glc (2-1)Glc	Glc (6-1) Araf
	Rd	Glc (2-1)Glc	Glc
	Rg3	Glc (2-1)Glc	H
	Ra1	Glc (2-1)Glc	Glc-Arap-Xyl
	Ra2	Glc (2-1)Glc	Glc-Araf-Xyl
Protopanaxatriol ginsenosides PPT	Rg1	Glc	Glc
	Rg2	Glc (2-1)Rha	H
	Re	Glc (2-1)Rha	Glc
	Rf	Glc (2-1)Glc	H
	Rh1	Glc	H
Oleanolic acid ginsenosides	Ro	GlcUA (2-1)glc	Glc

## Pharmacological effects of ginseng polysaccharides in treating cardiovascular diseases

There are many kinds of polysaccharides which are widely distributed. They can be divided into extracellular and intracellular polysaccharides. Among them, plant polysaccharides and microbial polysaccharides are more studied (Jin and Zhang, 1995). In recent years, ginseng polysaccharides have been paid more and more attention as an important component of ginseng to exert pharmacodynamic activity, and ginseng polysaccharides with different structural characteristics and activities have been reported widely. Ginseng polysaccharides are mainly divided into two categories: neutral sugar and acidic pectin (Li and Zhang, 1986). The main active ingredient is ginseng pectin, which is mainly composed of galactosyl, galacturonic acid, arabinose, and rhamnosyl (Zhang et al., 1982; Li and Zhang, 1984). Ginseng polysaccharide has high antioxidant activity, can significantly scavenge hydroxyl radicals and superoxide anions, its mass concentration has a certain dose-effect relationship with antioxidant activity, and is a good natural antioxidant (Zhou et al., 2015). Studies have shown that ginseng polysaccharides can improve oxidative stress injury in cardiomyocytes by inhibiting ROS and apoptosis (Tian et al., 2018). Another study determined the antioxidant activity of ginseng polysaccharides, and the results showed that the antioxidant activity of neutral polysaccharides was higher than that of acid polysaccharides in the aboveground part of ginseng, while the antioxidant activity of acid polysaccharides in the underground part of ginseng was not large (Chen and Huang, 2019). In addition, ginseng polysaccharide can improve its energy metabolism disorder and increase the vitality of mitochondria (Zhang et al., 2013b). A short review proposed that Rb1 can regulate mitochondrial energy metabolism, mitochondrial fission and fusion, apoptosis, oxidative stress and reactive oxygen species release, mitophagy and mitochondrial membrane potential (Zhou et al., 2019). Other studies have found that ginseng polysaccharides can regulate the activities of GSH and SOD enzymes, significantly reduce the expression levels of B cell lymphoma-2 (Bcl-2) and Bcl-2 Assaciated X protein (Bax) in rats, and improve dyslipidemia in rats with coronary heart disease (Wan et al., 2020).

## Pharmacological effects of ginseng volatile oil in treating of cardiovascular disease

Ginseng volatile oil has the special aroma of ginseng. Ginseng stems, leaves and flowers have higher level of volatile oils, while ginseng roots have less volatile oils (Chen et al., 1982). The identification of ginseng volatile oil found that there are many more terpenes (Zhang et al., 1994), followed by oxygenated compounds and long-chain alkanes (Sun and Wang, 1997). Volatile oils include compounds such as pentadecane and n-hexadecanoic acid, etc (Yan et al., 1994; Wu et al., 1996). The results of cell experiments suggest that ginseng volatile oil can inhibit the secretion of inflammatory factors such as TNF-α, IL-6 and IL-1β, and inhibit the NF-κB pathway, thereby playing an anti-inflammatory effect (Zuo, 2021). Ginseng volatile oil also has obvious protective effect on ischemic myocardial injury in animals, and can improve blood rheology, anti-platelet aggregation, reduce blood viscosity, and prevent thrombosis (Teng et al., 1989; Zhang, 2016). Ginseng volatile oil can be used for the treatment of coronary heart disease and angina pectoris. It can reduce the content of cardiac troponin I (cTn-I) in serum, reduce the content of Malondialdehyde (MDA), increase the activity of SOD and GSH enzymes, increase the concentration of NO, and protect the myocardium through the mechanism of anti-oxidative damage (Chen, 2015).

Although many studies have proved that ginseng volatile oil has good efficacy on cardiovascular diseases, a recent review concluded that the research on ginseng volatile oil is still in its infancy due to the limitation of the production process (Chen et al., 2022). More large samples and high-quality studies are needed to verify the efficacy of ginseng volatile oil in the treatment of cardiovascular diseases.

## Clinical study on the treatment of cardiovascular diseases with ginseng prescriptions

A meta-analysis study using nitrate as a control drug found that ginseng-based drug had a significant effect on symptomatic improvement of angina pectoris and improvement in electrocardiogram (Jia et al., 2012). Another study focused on the efficacy of ginseng prescription in the treatment of patients with coronary angina pectoris, and the study showed that the patients who took the ginseng prescription had greater improvement in ECG results, clinical symptoms and nailfold microcirculation (Yuan et al., 1997). It can be seen that the important role of ginseng-containing prescriptions in the prevention and treatment of cardiovascular diseases should not be ignored. The mechanic and clinical research progress of related drugs is summarized as follows (Table 2).

**TABLE 2:** Mechanism and Clinical Indicators of Ginseng Prescriptions in treating Cardiovascular Diseases.

Ginseng prescriptions	Diseases	Mechanism	Clinical indicators
Xin-su-ning capsule	Tachyarrhythmias atrial fibrillation	prolong APD and increase ERP of cardiac electrical conduction	improve the total effective rate of holter, reduce premature ventricular contractions and the frequency of atrial fibrillation
Yi-xin-shu capsule	angina pectoris diastolic heart failure	lower lipids and anticoagulation, inhibit platelet adhesion and aggregation, improve blood rheology	improve the incidence frequency and degree, echocardiographic indicators, 6-min walking test results, QOL
Tong-xin-luo capsule	unstable angina pectoris myocardial infarction	decrease vWF and Fn, improve blood lipid and hypercoagulability, reduce blood viscosity and level of IL-18, hs- CRP and CK	improve the function of ischemic myocardium, reduce the scope of myocardial infarction
Qili Qiangxin capsule	chronic heart failure	increase ATP, ADP, eNOS and p-AMPK, activate AMPK-eNOS pathway, reduce ICAM-1	increase CO, reduce LVEDP, LVEDD, LVEF and 6-min walk distance, improve exercise tolerance and cardiac function
Qishen capsule	angina pectoris	reduce blood lipids, blood viscosity and fibrinogen, the levels of plasma hs-CRP and BNP	reduce the frequency and myocardial oxygen consumption of angina pectoris, improve the electrocardiogram and cardiac function
Shenshao capsule	unstable angina pectoris myocardial remodeling	reduce CRP, cTnI, IL-6, NO, ET and tPAI-1, decrease WBV, PV, FIB, PAR, TC, TG and LDL-C	reduce the number of carotid plaques, the plaque volume, the cardiac index and arterial intima-media thickness
Shexiang Baoxin pill	ischemic myocardium stable angina pectoris hyperlipidemia	increase VEGF, bFGF, FVIII, SOD, decrease TC, LDL-C	reduce the infarction size of ischemic myocardium, the hyperplasia of arterial intima, decrease all-cause deaths, heart failure events, stroke events
Shensong yangxin capsule	arrhythmia	block IK1, Ito, delay Ik	improve cardiac function, shorten the effective refractory period, reduce the TDR

### Xin-su-ning capsule

Xinsuning Capsule can be used to treat tachyarrhythmias. Compared with propafenone and mexiletine hydrochloride, it can effectively improve the total effective rate of holter in patients with premature ventricular contractions, reduce premature ventricular contractions (Wang and Lu, 2008), and significantly improve the clinical symptoms of patients (Yuan, 2000; Song et al., 2022). The frequency of atrial fibrillation was significantly reduced and the incidence of adverse reactions was lower after the addition of Xinsuning Capsules on the basis of conventional western medicine treatment (Li and Zhang, 2015). Xinsuning capsules combined with low-dose betaloc in the treatment of premature ventricular contractions can significantly reduce the number of premature ventricular contractions, maintain ventricular muscle stability, and improve vascular endothelial function and clinical symptoms (Yang, 2020). It is worth noting that Xinsuning can prolong the action potential duration (APD) and increase the effective refractory period (ERP) of cardiac electrical conduction, thereby inhibiting reentry-induced arrhythmias, and has a good antiarrhythmic effect (Jiao and Ding, 2015).

### Yi-xin-shu capsule

Yixinshu Capsule is widely used clinically in coronary heart disease, angina pectoris and chronic heart failure. The capsule has the functions of lowering lipids and anticoagulation, inhibiting platelet adhesion and aggregation, and improving blood rheology (Li and Shi, 2013; Wang and Zhi, 2016). Yixinshu Capsule can also improve the symptoms of shortness of breath, fatigue and dry mouth (Cao, 2009; Cao and Xie, 2010). On the basis of conventional western medicine combined with Yixinshu Capsule in the treatment of diastolic heart failure, it was found that the echocardiographic indicators, 6-min walking test results, the quality of life (QOL)were improved, and were better than those of simple western medicine treatment (Zhang and Zhu, 2011). In the treatment of unstable angina pectoris (UA) of CHD, Yixinshu Capsule is better than the control group which only given antiplatelet aggregation and anticoagulant drugs on the frequency of angina pectoris, the degree of angina pectoris and the electrocardiogram (Tao et al., 2009).

### Tong-xin-luo capsule

After the application of Tongxinluo Capsules to treat patients with unstable angina pectoris, the plasma Von Willebrand factor (vWF) and fibronectin (Fn) decreased, which can protect vascular endothelial cells (Xiao et al., 2002). Tongxinluo Capsules can also improve the function of ischemic myocardium in patients undergoing percutaneous coronary intervention (PCI) or thrombolysis after acute myocardial infarction (AMI), and can restore the function of part of the viable myocardium, improve myocardial remodeling after AMI (You et al., 2004), significantly improve blood lipid metabolism, reduce blood viscosity, and improve blood hypercoagulability (Wu et al., 2001). Studies have shown that the addition of Tongxinluo Capsules on the basis of western medicine treatment can improve the clinical efficacy of angina pectoris in elderly patients with coronary heart disease, and reduce the serum levels of interleukin-18 (IL-18) and high-sensitivity C-reactive protein (hs- CRP) level (Wang and Li, 2012). Experimental studies have also found that Tongxinluo Capsule can reduce the scope of myocardial infarction after ischemia-reperfusion in rats, reduce the level of plasma CK, reduce the degree of myocardial necrosis, and have a protective effect on ischemia-reperfusion myocardium (Zhao et al., 2000).

### Qili Qiangxin capsule

Studies have found that Qili Qiangxin Capsule can significantly increase the left ventricular myocardial contractility and cardiac output (CO), reduce left ventricular end-diastolic pressure (LVEDP), at the same time can increase renal blood flow, effectively improve cardiac function (Liu et al., 2007). Another study found that Qili Qiangxin Capsule can significantly reduce the left ventricular end-diastolic diameter (LVEDD) in patients with chronic heart failure, reduce the plasma vasopressin (AVP) concentration, significantly increase the plasma brain natriuretic peptide (BNP), left ventricular ejection fraction (LVEF) and 6-min walk distance, improve exercise tolerance (Huang, 2010; Wu et al., 2011; Chen and Zhou, 2015). Qili Qiangxin Capsule can significantly increase the content of ATP and ADP in myocardial tissue, reduce the expression of ICAM-1 mRNA, increase eNOS mRNA, and significantly increase the protein expression of p-AMPK. It is suggested that Qiliqiangxin can protect the myocardial capillary endothelium in rats with pressure overload, and its mechanism may be related to the activation of AMPK-eNOS pathway (Zhang et al., 2013c).

### Qishen capsule

Qishen Capsule is one of the effective drugs for the treatment of coronary heart disease angina pectoris. It can improve clinical symptoms, reduce blood lipids, blood viscosity and fibrinogen (Shang, 2003), and can also reduce the levels of plasma hs-CRP and BNP (Deng and Liu, 2013), the frequency of angina pectoris and the rate of vasodilator drugs. It also has a significant effect on patients with myocardial infarction complicated by cardiac insufficiency and coronary artery bypass grafting (Wang, 2010), (Yang and Huang, 2013). On the basis of nicorandil as the control group, combined with Qishen Capsule can improve the total effective rate of patients with coronary heart disease, reduce the frequency and myocardial oxygen consumption of angina pectoris, and improve the electrocardiogram and cardiac function indicators (Li et al., 2022).

### Shenshao capsule

Shenshao Capsule can reduce the frequency and shorten the duration of angina attacks, and reduce the levels of serum CRP, cTnI and interleukin-6 (IL-6) in patients with unstable angina pectoris. It also has a decreasing effect on indicators such as whole blood viscosity (WBV), plasma viscosity (PV), fibrinogen (FIB) and platelet adhesion rate (PAR) (Liu et al., 2020b). Shenshao Capsule not only has a significant clinical effect in the treatment of coronary heart disease, but also can reduce the dosage of nitroglycerin and the blood lipid level of patients. After treatment, the levels of TC, TG and LDL-C were decreased, and HDL-C was increased (Zhang and Ding, 2021). Because of this, Shenshao Capsule can also reduce the number of carotid plaques, the plaque volume, and arterial intima-media thickness (IMT) (Li, 2015). After using Shenshao Capsule, the cardiac index was reduced, and the levels of nitric oxide (NO), endothelin (ET) and tissue plasminogen activation inhibitor (tPAI-1) in myocardium are both decreased (Li et al., 2010).

### Shexiang Baoxin pill

Shexiang Baoxin Pill can significantly reduce the infarction size of ischemic myocardium, and there is a dose-effect relationship (Wang et al., 2004). The expression levels of vascular endothelial growth factor (VEGF), basic fibroblast growth factor (bFGF), factor VIII and the surface density of blood vessels in the infarction edge area were significantly increased, suggesting that Shexiang Baoxin Pill can promote the angiogenesis of coronary collaterals (Wang et al., 2002). A prospective, randomized, non-blind controlled clinical trial involving 200 patients with stable angina pectoris also showed that after the treatment of Shexiang Baoxin Pill, the use of nitrates in the treatment group was significantly lower than that before treatment, all-cause deaths, heart failure events, and stroke events also tended to decrease (Zhu et al., 2010). Shexiang Baoxin Pill can also reduce the damage of hyperlipidemia to the artery. It can significantly inhibit the rise of serum total cholesterol (TC) and low-density lipoprotein cholesterol (LDL-C) level and increase the concentration of serum SOD. Reduce the hyperplasia of arterial intima, blood damage to the arterial wall, and inhibit the formation of atherosclerosis (Luo et al., 1998).

### Shensong Yangxin capsule

Shensong Yangxin Capsule has a broad-spectrum antiarrhythmic effect, and can block inward rectifier potassium current (IK1), instantaneous outward potassium current (Ito) and delayed rectifier potassium current (Ik) in cardiomyocytes to varying degrees. Importantly, Shensong Yangxin Capsules have fewer proarrhythmic side effects (Li et al., 2007), and may also significantly improve clinical symptoms such as palpitation, insomnia, shortness of breath and fatigue in patients with arrhythmia (Chen et al., 2006; Bai and Wang, 2011). In addition, Shensong Yangxin Capsule can play an anti-arrhythmic effect after heart failure, because it can improve cardiac function, and at the same time, it can not only shorten the effective refractory period of the left and right ventricles, but also reduce the transmural dispersion of repolarization (TDR) (Wang et al., 2012).

## Adverse reactions of ginseng medication

Although ginseng has a wide range of clinical applications, it should not be abused. The pharmacopoeia stipulates that the single dose of ginseng is 3–9 g. Taking the recommended dose of ginseng will not cause serious adverse reactions. Studies have shown that high doses of ginseng (15 g/d) will lead to ginseng abuse syndrome (GAS), the specific performance is as follows (Siegel, 1979): ①Nervous system: headache, dizziness, fever, restlessness, easy to wake up, insomnia, sweating, euphoria, mania, confusion, cerebral arteritis, mydriasis. ②Cardiovascular system: arrhythmia, palpitations, slow heart rate, high blood pressure, and even heart failure. ③Endocrine and metabolic system: hypokalemia, gynecomastia, breast pain. ④Blood system: neutropenia, gastrointestinal bleeding, uterine bleeding, cerebral hemorrhage. ⑤Digestive system: abdominal pain, nausea and vomiting, intractable. ⑥Respiratory system: shortness of breath, asthma.

In addition, ginseng will also have an effect on other drugs, and it is particularly important to pay attention to clinical use (Ryu and Chien, 1995). There are literature reports of interactions between ginseng and warfarin, which inhibits the pharmacological effects of warfarin and may increase the risk of blood clotting (Smolinske, 1972; Janetzky and Morreale, 1997). Studies have found that ginseng has an inhibitory effect on monoamine oxidase, similar to monoamine oxidase inhibitors (MAOIs), and should be clinically cautiously combined with antidepressants such as phenylcyprotamine and phenelhydrazine (Dai and Yin, 1987). Studies have reported that ginseng can be used only for mild diabetic patients, for moderate and severe patients, when people participate in the combination of insulin or oral hypoglycemic drugs, it will have a synergistic effect, which may lead to low glucose, and the dose of hypoglycemic drugs needs to be reduced (An et al., 2003).

## Summary and prospect

This paper mainly focus on the mechanism of ginsenosides, ginseng polysaccharides, and ginseng volatile oil, summarizes the progress of ginseng-containing drugs, lists the adverse reactions of ginseng medication, and expounds the multi-faceted effects of ginseng functional compounds and composition compatibility in cardiovascular diseases. According to the existing research, ginseng and ginseng-containing drugs can treat coronary heart disease by improving inflammation and lowering blood lipid levels, can inhibit the activation of glycoprotein IIb/IIIa in human platelets, it can increase coronary perfusion flow and promote capillary regeneration by antioxidant, anti-inflammatory and anticardiomyocyte apoptosis. Ginseng can also reduce heart rate, lower action potential, and suppress arrhythmias by activating K^+^ channels, blocking Ca^2+^ channels and Na^+^ currents. By improving antioxidant enzyme function, increasing energy metabolism and reducing free radical damage, ginseng can inhibit heart failure and ventricular remodeling. Although the role of ginseng on blood pressure has been controversial in the past, recent studies have shown that ginseng can lower blood pressure by promoting endothelial-dependent vasodilation. It can be seen that ginseng has a good efficacy as a drug for the treatment of cardiovascular diseases and can play a therapeutic role through multiple pathways, which is worth continuing research and development. Besides, there are many related researches on ginsenosides, but relatively few researches on ginseng polysaccharides and volatile oils. Due to the various and complex components of ginseng volatile oils, there are still many unknown unique components of ginseng volatile oils to be separated, identified and developed. At present, there is a lot of clinical evidence for ginseng-containing medicines, but further meta-analysis and quality evaluation are needed to reasonably and clearly explain its therapeutic effects and help the innovative application of botanical medicine.

In order to better explore the natural Chinese herbal medicine represented by ginseng, further develop its functional components, and improve its role in the treatment and prevention of diseases, the following aspects can be achieved: ① Use the theory of traditional Chinese medicine to expand the efficacy and indications of Chinese herbal medicine. Ancient Chinese medicine books are the crystallization of thousands of years of medical practice experience. Tu Youyou was inspired to develop artemisinin from a medical treatise by Ge Hong of the Eastern Jin Dynasty (317–420). Medicine tailored from classic Chinese medicine recipes also play an important role in the fight against COVID-19. ②Pay attention to the combination of ginseng medication. The composition of traditional Chinese medicine prescription is not a simple combination of several drugs, and the rational compatibility and application of rules can improve the clinical efficacy of ginseng. ③Adjust the dosage of Chinese herbal medicine according to the difference of symptoms. The therapeutic effect of Chinese herbal medicine has a dose-effect relationship, and the optimal dose should be selected according to the disease. Taking ginseng as an example, small doses of ginseng are suitable for healthcare people, which can improve physical fitness and enhance disease resistance. Patients with chronic diseases are suitable for medium doses of ginseng, and patients with massive hemorrhagic shock are suitable for large doses of ginseng. ④ Improve the preparation technology of Chinese herbal medicine to promote the development of the industry. There are various types of Chinese herbal preparations in ancient times. In modern times, it is necessary to further use new technologies and methods to design drug delivery systems based on biopharmaceutical characteristics, and to improve the absorption and bioavailability of functional compounds in traditional Chinese medicine preparations, so as to better exert its pharmacological effects.
